# Fast and Sensitive Detection of SARS-CoV-2 Nucleic Acid Using a Rapid Detection System Free of RNA Extraction

**DOI:** 10.1155/2023/8053524

**Published:** 2023-01-20

**Authors:** Liang Ma, Yanyan Fan, Xiaomu Kong, Yongwei Jiang, Hong Huang, Meimei Zhao, Yi Liu, Peng Gao, Yong Cui, Yongtong Cao

**Affiliations:** ^1^Department of Clinical Laboratory, China-Japan Friendship Hospital, Beijing 100029, China; ^2^Department of Pulmonary & Critical Care Medicine, Laboratory of Clinical Microbiology & Infectious Diseases, China-Japan Friendship Hospital, Beijing 100029, China; ^3^Coyote Bioscience USA Inc., 965, Atlantic Ave, Suite 101, Alameda, CA 94501, USA; ^4^Department of Dermatology, China-Japan Friendship Hospital, Beijing 100029, China

## Abstract

**Objectives:**

To establish and evaluate the analytical and clinical performance of the Flash20 SARS-CoV-2 nucleic acid rapid detection system free of RNA extraction.

**Methods:**

The limit of detection (LoD) was determined using a negative nasopharyngeal swab matrix spiked with different concentrations of SARS-CoV-2 virus; a total of 734,337 reference sequences of viral genomes from GenBank were used for the in-silico analysis to assess the inclusivity of the assay. The specificity of the system was evaluated by testing 27 medically relevant organisms. A total of 115 clinical specimens were collected and tested on the Flash20 SARS-CoV-2 detection system and with an FDA-approved comparator test to assess the clinical performance of the system.

**Results:**

The LoD of the Flash20 SARS-CoV-2 detection system is 250 copies/mL with a positive rate ≥90% (*n* = 20); alignments results showed that over 99% identity of the primer and probe of the Flash20 SARS-CoV-2 nucleic acid rapid detection system to the available SARS-CoV-2 sequences; the omicron samples tested 100% positive. None of the 27 organisms showed cross-reactivity with the Flash20 SARS-CoV-2 nucleic acid rapid detection system. Among all the 215 clinical samples, the Flash20 SARS-CoV-2 nucleic acid rapid detection system exhibits a high sensitivity of 99.24% (131/132) and 100% (83/83) specificity.

**Conclusion:**

The nucleic acid rapid detection system provides sensitive and accurate detection of SARS-CoV-2 free of RNA extraction. The high sensitivity and short time to results of approximately 35 minutes may impact earlier infection control and disease management.

## 1. Introduction

In December 2019, a cluster of patients with pneumonia of unknown cause was found in Wuhan, Hubei province in China. A novel betacoronavirus, severe acute respiratory syndrome coronavirus 2 (SARS-CoV-2), was identified as the causative agent and caused coronavirus disease 2019 (COVID-19) [1]. SARS-CoV-2 can be person-to-person transmitted effectively through aerosols or fomites [2, 3]. With transmission capabilities before symptom onset [4], this disease has rapidly spread across the world [5]. On 7th September, 2021, over 220 million of confirmed cases of COVID-19 had been reported globally and the cumulative number of deaths over 4.5 million [6]. The global public health issue caused serious economic losses worldwide. The World Health Organization (WHO) recommends a combination of measures to control the spread of COVID-19, including rapid diagnosis and immediate isolation of cases, rigorous tracking, and precautionary self-isolation of close contacts [7]. A fast, reliable, and accurate diagnostic test would play an extremely important role in SARS-CoV-2 infection prevention and control.

Nucleic acid amplification tests (NAATs), such as real-timereverse-transcription polymerase chain reaction (RT-qPCR), are the gold standard for diagnosing SARS-CoV-2 infection [8–10]. The National Medical Products Administration (NMPA) in China has approved RT-qPCR technology as detection method for COVID-19 [11]. Conventional RT-PCR is a multistep process that involves the isolation and purification of nucleic acids from a clinical sample and detects the viral RNA. It is tedious, time-consuming, labor-intensive, and requires highly skilled technicians. In this case, the SARS-CoV-2 tests have required turnaround times of nearly 6–8 hours or more [12, 13]. The use of testing with a rapid turnaround may allow for an earlier detection isolation of confirmed cases, facilitate earlier infection control, and disease management [13, 14].

**Figure 1:**
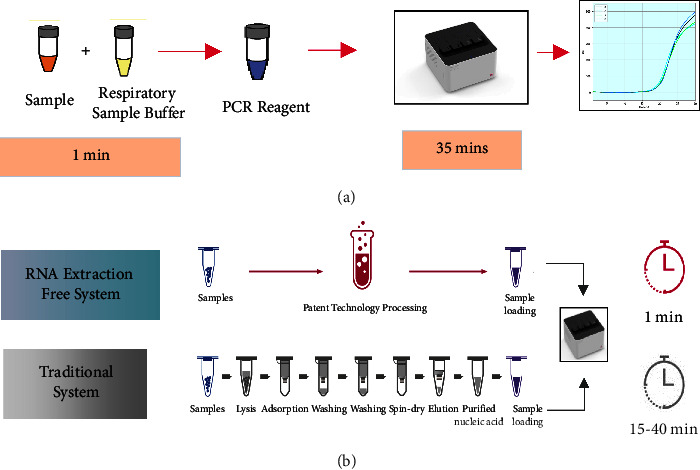
SARS-CoV-2 POC test processing. (a) Schematic diagram of real-time RT-PCR-based SARS-CoV-2 POC test without the need for RNA extraction. (b) Extraction-free technology reduced a normally 6- to 8-step procedure down to a 2-step process.

In this study, we developed a real-time RT-PCR-based SARS-CoV-2 POCT (Point of Care Test) (Coyote Bioscience Co., Ltd., Beijing, China) that can be performed without the need for RNA extraction and uses one-step, real-time RT-PCR directly on a fast real-time PCR cycler (Figure 1). The entire process of sample to result can be completed within 35 minutes, which is much quicker than the 3-4 hours required for conventional real-time RT-PCR. The rapid SARS-CoV-2 detection system is evaluated for the analytical performance, including the limit of detection (LoD), inclusivity, cross-reactivity, and interference. The clinical performance of the system was assessed by testing 115 clinical samples and compared with the DiaCarta QuantiVirus™ SARS-CoV-2 Test Kit, which is FDA EUA-authorized.

## 2. Materials and Methods

### 2.1. Reagent and Instrument

Flash20 SARS-CoV-2 nucleic acid rapid detection system is developed based on the technical of molecular parallel reaction. The nucleic acid of the virus is released by the lysis agent prior to or during the process of reverse transcription amplification in parallel with deoxyribonucleic acid (DNA) amplification. The system is RNA extraction-free, includes inhibition-resistant reverse transcriptase and DNA polymerase, and includes the addition of a PCR enhancer, which improves amplification efficiency and leads to the overall higher sensitivity of the assay. Briefly, a 15 *μ*L oropharyngeal or nasopharyngeal swab sample in Coyote's VTM is treated by 15 *μ*L of respiratory sample buffer. The treated sample can be tested by adding to RT-PCR reaction directly. The thermal cycles are as follows: 42°C for 3 min, 94°C for 10 s (94°C for 3 sec and 55°C for 10 sec)^*∗*^ 15 cycles, (94°C for 3 sec, 55°C for 10 sec, and reading)^*∗*^ 30 cycles. The entire testing time from sample to answer is about 35 min.

DiaCarta QuantiVirus™ SARS-CoV-2 Test Kit was used as a reference reagent in the clinical evaluation. DiaCarta kit is conducted on ABI7500 real-time PCR instrument.

### 2.2. Limit of Detection (LoD)

The LoD was determined by evaluating different concentrations of SARS-CoV-2 virus from BEI Resources spiked into SARS-CoV-2 negative nasopharyngeal matrix. The negative nasopharyngeal matrix is mixed with 10 negative nasopharyngeal swabs eluted with Coyote viral transport medium. A total of 6 concentrations, including 2000 copies/mL, 1000 copies/mL, 500 copies/mL, 400 copies/mL, 300 copies/mL, and 200 copies/mL were tested. The lowest concentration at which all 4 replications were positive was designated as the tentative LoD. A panel of 5 concentrations around the tentative LoD, including 300 copies/mL, 250 copies/mL, 200 copies/mL, 150 copies/mL, and 100 copies/mL were further tested in replicates of 20 to determine the LoD. The LoD is the lowest concentration that can be reproducibly detected ≥90% of the time.

### 2.3. Inclusivity Analysis

Evaluation of the reactivity of the Flash20 SARS-CoV-2 nucleic acid rapid detection system was performed by sequence alignment of the assay oligonucleotide primer and probe sequences with all publicly available nucleic acid sequences for SARS-CoV-2 in GenBank as of March 29, 2021, to demonstrate the predicted inclusivity of the Flash20 SARS-CoV-2 nucleic acid rapid detection system. The genomes enrolled for in-silico analysis met the following criteria: (1) genome length > 29,000 bp; (2) N proportion (not specific A, T, C, and G) <5% of the genome; (3) isolated from a human source.

The cultured Omicron variant was validated for inclusivity by spiking the variant into negative nasopharyngeal matrix to the concentration of 750 copies/mL. The Omicron variant samples were tested by the Flash20 SARS-CoV-2 nucleic acid rapid detection system for three replicates.

### 2.4. Specificity Analysis

A total of 27 organisms were obtained and tested to evaluate the specificity of the kit. Human coronavirus 229E, human coronavirus NL63, MERS-coronavirus (irradiated), adenovirus, human metapneumovirus (hMPV), human parainfluenza virus 1, human parainfluenza virus 2, human parainfluenza virus 3, human parainfluenza virus 4a, human parainfluenza virus 4b, influenza B, enterovirus, and respiratory syncytial virus A were from BEI resource, and human coronavirus OC43, influenza A, Rhinovirus, *Haemophilus influenzae*, *Streptococcus pneumoniae*, *Streptococcus pyogenes*, pooled human nasal wash, *Bordetella pertussis*, *Mycoplasma pneumoniae*, *Chlamydia pneumoniae*, *Legionella pnuemophila*, *Staphylococcus aureus*, *Staphylococcus epidermidis*, and *Candida albicans* were obtained from ATCC. Three replicates of each organism stock were tested.

### 2.5. Interference Study

SARS-CoV-2 negative nasopharyngeal swabs were collected in triplicate and spiked with potential interferent, including nasal secretion, dexamethasone, zanamivir, tobramycin, adrenalin, menthol, and blood (human). Additional negative nasopharyngeal swabs were collected and spiked with SARS-CoV-2 inactive virus (CoA NR-52287 BEI resource, lot number 70039068) at 3X LoD in addition to the potential interfering substances. Three concentration levels of the interference substances were tested in triplicates.

### 2.6. Clinical Evaluation

The clinical performance study was conducted with 179 leftover samples and 36 fresh samples collected and tested by PacGenomic CLIA Lab (Part I), DiaCarta CLIA Lab (Part II), and the China-Japan Friendship Hospital. A consent form was signed by the patients of these samples that agreed their sample can be used for investigational studies. Among these patients, 108 were male and 105 were female and the gender of the other 2 was unknown. The number of patients aged between 25 and 64 were 139 in total. 115 samples were collected at PacGenomic lab and DiaCarta CLIA Lab and tested for their SARS-CoV-2 infection status with the TaqPath RT-PCR assay. The samples were kept frozen for up to 8 weeks. 100 samples were collected at the China-Japan Friendship Hospital. 64 of these samples were kept frozen for up to 6 weeks, and 36 of these samples were fresh sample. The samples were chosen randomly and blinded to the operators to be tested on the new system. These leftover samples were split into two parts, one part was tested on the Flash20 FlashDetect™ SARS-CoV-2 Detection system and second on the comparator test (DiaCarta QuantiVirus™ SARS-CoV-2 Test Kit, which is FDA-authorized EUA with the LoD of 600 NDU/mL. The sample information is provided in Tables 1 and 2.

### 2.7. Statistical Analysis

A two-by-two table was constructed to assess the agreement between the Flash20 SARS-CoV-2 nucleic acid rapid detection system and the DiaCarta QuantiVirus™ SARS-CoV-2 Test Kit. The level of agreement between assays was determined by Cohen's kappa, the sensitivity (positive percent agreement) and specificity (negative percent agreement) with two-side 95% confidence interval (CI).

## 3. Results

### 3.1. The LoD for Nasopharyngeal/Oropharyngeal Swabs

**Figure 2:**
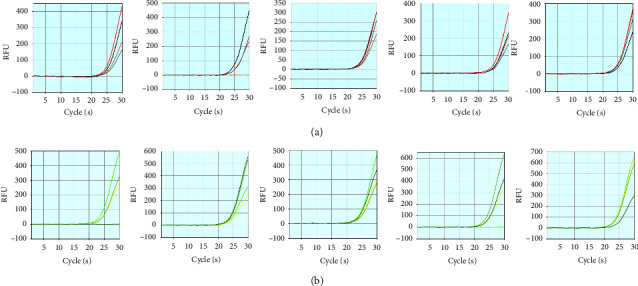
PCR amplification curve of SARS-CoV-2 LoD sample by spiking the SARS-CoV-2 virus into the negative matrix to 250 copies/mL. (a) The ORF1ab amplification curve for 20 replicates. (b) N gene amplification curve for 20 replicates.

LoD studies were performed to determine the lowest detectable concentration of SARS-CoV-2 at which approximately 95% of all (true positive) replicates are tested positive. The detection rate of the system for virus samples no less than 300 copies/ml is 100%. When the virus concentration is more than 250 copies/ml, the detection rate of the Flash20 SARS-CoV-2 nucleic acid rapid detection system for positive samples is more than 95% (95%-ORF1ab, 90%-N). The results of the LoD confirmation study are shown in Table 3. The Ct values of the LoD estimation are provided in Appendix-1. These results demonstrate that the LoD of the Flash20 SARS-CoV-2 nucleic acid rapid detection system is 250 copies/mL for nasopharyngeal/oropharyngeal swabs, which is 1.875 copies/reaction. The amplification curves for ORF1ab and N genes of SARS-CoV-2 with a concentration of 250 copies/mL are shown in Figure 2.

### 3.2. Inclusivity Evaluation Results

A total of 4,086,522 isolates that met enrollment criteria were enrolled. The 4,086,522 viral genomes were aligned against the primer/probe sets used.

**Figure 3:**
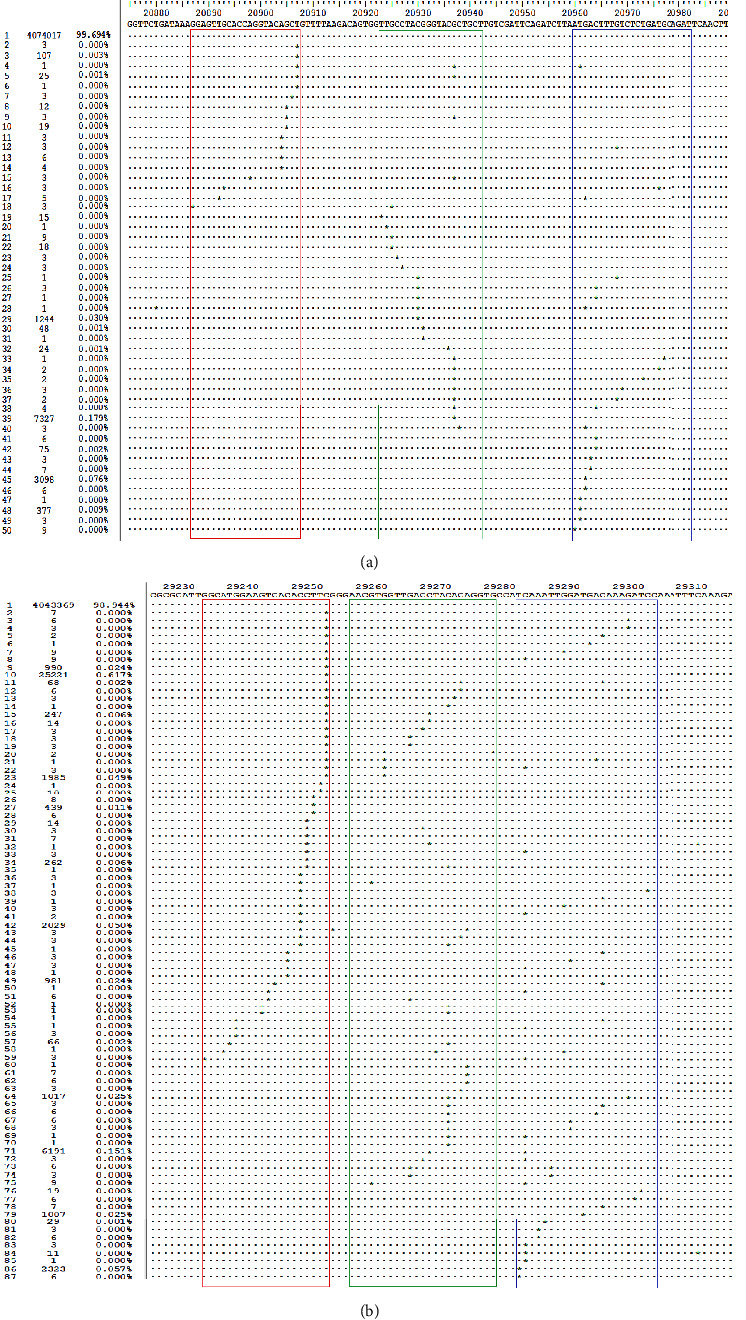
In-silico analysis of primer and probe of ORF1ab and N gene with SARS-CoV-2 sequence from GISAID database. (a) In-silico analysis result of primer and probe of ORF1ab. (b) In-silico analysis result of primer and probe of N gene. The symbol. presents the consistent sequence, and ^*∗*^ presents the mismatch base pair. The forward primer is marked by red box, probe marked by green box, and reverse primer marked by blue box.

All of the alignments showed over 99% identity of the Flash20 SARS-CoV-2 nucleic acid rapid detection system to the available SARS-CoV-2 sequences. The sequences containing more than 2 mismatches in primer and probe and more than 1 mismatch in the 3′ end of primer and probe are listed in Figure 3.

**Figure 4:**
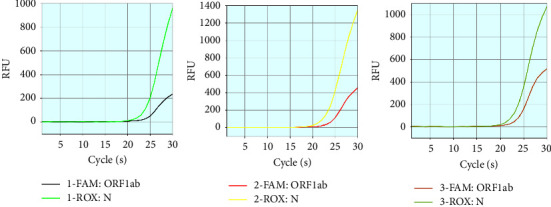
PCR amplification curve of Omicron variant sample with the concentration of 750 copies/mL.

The Omicron variant sample with the concentration of 750 copies/mL was tested 100% positive. The amplification curve for ORF1ab and N gene of the Omicron variant sample is listed in Figure 4.

### 3.3. Specificity Analysis

All 27 organisms evaluated gave negative results for the detection of the SARS-CoV-2 virus, which demonstrates that the Flash20 SARS-CoV-2 nucleic acid rapid detection system assay design does not react with related pathogens or other highly prevalent disease agents. The results of the specificity analysis are shown in Table 4.

### 3.4. Interference Study Results

None of these potential interferences were found to inhibit the performance of the assay, as all swabs, with and without the potential interferences were found to have the expected results. The results of the interference study results are shown in Table 5.

### 3.5. High Sensitivity and Specificity for Clinical Samples

Among the total of 115 clinical samples, 63 were tested at the PacGenomic site, 52 were tested at DiaCarta site, and the rests were tested at the China-Japan Friendship Hospital. The positive percent agreement and negative percent agreement between the FDA Emergency Use Authorized RT-PCR test and the Flash20 SARS-CoV-2 nucleic acid rapid detection system were calculated using all the valid results. As indicated in Table 6, the Flash20 SARS-CoV-2 nucleic acid rapid detection system exhibits a high sensitivity and specificity of 99.24% (131/132) and 100% (83/83), respectively. Individual sample results on the Flash20 FlashDetect™ SARS-CoV-2 Detection kit and the DiaCarta QuantiVirus™ SARS-CoV-2 are provided in Appendix-2.

### 3.6. Comparison with Other COVID-19 Nucleic Acid System

The performance of Flash20 SARS-CoV-2 detection system was compared with three SARS-CoV-2 detection systems approved by the U.S. Food and Drug Administration (FDA), and the information is listed in Table 7.

## 4. Discussion

In this study, we showed a rapid detection system for nucleic acid from the SARS-CoV-2 virus, with high sensitivity and specificity. The Flash20 SARS-CoV-2 system is an easy-to-use, on-demand format that generates results in about 35 min.

Current routine PCR products for diagnostics for SARS-CoV-2 can perform high-throughput processing, but the turnaround time limits their usability in patient management and infection control. The use of rapid diagnostic tests with sensitivity and specificity comparable with current standard molecular diagnostic because of the reduced time spent by uninfected individuals in health-care settings where they may be at increased risk of infection. Lessons learned from the recent Ebola virus and Zika virus epidemics are that delay in developing the right diagnostic for the right population at the right time has been a costly barrier to disease control and prevention [15]. If rapid tests had been available throughout the Ebola epidemic, one study estimate, for Sierra Leone, fast detection testing might have reduced the scale of the epidemic by over a third [16].

Key advantages of rapid molecular diagnostic products are simple operation procedures and rapid detection, which is significantly faster than the seven hours currently required by traditional molecular testing. High-speed detection reduces turnaround time (TAT) for the diagnosis of COVID-19, thus allowing prompt decision making regarding the isolation of infected patients [17]. Multiple rapid molecular diagnostic products have received FDA emergency use authorization (EUA), including Cepheid Xpert Xpress SARS-CoV-2 (Xpert Xpress) and Abbott ID NOW COVID-19 (ID NOW) [18]. Xpert Xpress is easy to use and the run-time is 45 min, which include loading the sample and the cartridge. ID NOW has the sample-to-answer time at about 17 min to result. Marie et al. compared Xpert Xpress and ID NOW to the Roche Cobas SARS-CoV-2 assay for samples with low, medium, and high viral concentrations [19]. The Xpert Xpress showed a very high level of agreement with the cobas assay, but ID NOW did not detect most specimens with Ct value ≥30. These findings confirm those published by Hogan et al. [20–22]. In this study, we assess the analytical and clinical performance of Flash20 SARS-CoV-2 system. Flash20 SARS-CoV-2 system is a rapid, sensitive, and accurate platforms with results available in 35 min, and hands on time about 1-2 min. Flash20 SARS-CoV-2 nucleic acid rapid detection system is developed based on direct RT-qPCR with simple sample treatment. Proper specimen collection and storage are critical to the performance of this test. PCR inhibitors should be avoided in RT-qPCR reactions. Synthetic fiber swabs with thin plastic or wire shafts were recommended by the Centers for Disease Control and Prevention (CDC). Calcium alginate swabs or swabs should not be used with wooden shafts, as they may contain substances that inactivate some viruses and may inhibit molecular tests. As guanidine-contained VTM is incompatible with RT-qPCR, VTM without guanidine should be used with the Flash20 SARS-CoV-2 nucleic acid rapid detection system. Coyote's VTM is validated and recommended to be use with the system. Insert a swab into the nostril, parallel to the palate, and leave the swab in place for a few seconds to absorb secretions. Place swab immediately into a sterile tube containing sample storage solution.

The LoD of the Flash SARS-CoV-2 system, established with SARS-CoV-2 virus in the nasopharyngeal matrix was 250 copies/mL. Fifty-four out of 55 positive samples detected by the reference kit (DiaCarta QuantiVirus™ SARS-CoV-2) were confirmed with the Flash20 SARS-CoV-2 nucleic acid rapid detection system, with a diagnostic sensitivity of 98.18%. The Flash20 SARS-CoV-2 system detected 18 low-level positive samples with Ct value >30, with the highest of Ct value equal 36.32. But it did not detect 1 low-level positive sample with Ct value of 34.03. The Flash SARS-CoV-2 system is for use on nasopharyngeal swabs in Coyote viral transport medium. The current evaluation on clinical samples was done using both DiaCarta and Coyote VTM, as choice of transport medium is limited during the current pandemic. Moreover, a 100% of analytical and clinical specificity was observed against other viruses, such as human coronavirus 229E, human coronavirus OC43, human coronavirus NL63, and MERS-coronavirus. The number of specimens included in the clinical trial is only 115, but these specimens were chosen to span the positivity range of clinical specimens, including those specimens with a low viral load.

The limitation of the Flash20 SARS-CoV-2 system is the small number of testing ports per instrument, since each instrument can support 4 positions. This limitation can be offset by the rapidity of the assay, and/or adding modules/bays for more capacity.

## Figures and Tables

**Table 1:** Patients' age distribution.

Patient age groups	All subjects (*n* = 215)
<14 years of age	29
14–24 years of age	38
25–64 years of age	139
≥65 years of age	9

**Table 2:** Genders of the patients.

Sex	All subjects (*n* = 215)
Male	108
Female	105
Unknown	2

**Table 3:** LoD determination study results.

Titer SARS-CoV-2 virus (copies/mL)	Replicates	ORF1ab gene positive result (%)	N gene positive result (%)	Percentage of positive rate (%)
2000	4	100 (4/4)	100 (4/4)	100 (4/4)
1000	4	100 (4/4)	100 (4/4)	100 (4/4)
500	4	100 (4/4)	100 (4/4)	100 (4/4)
400	4	100 (4/4)	100 (4/4)	100 (4/4)
300	4	100 (4/4)	100 (4/4)	100 (4/4)
200	4	75 (3/4)	75 (3/4)	75 (3/4)
300	20	100 (20/20)	100 (20/20)	100 (20/20)
250	20	95 (19/20)	90 (18/20)	95 (19/20)
200	20	80 (16/20)	85 (17/20)	95 (19/20)
150	20	80 (16/20)	80 (16/20)	90 (18/20)
100	20	50 (10/20)	60 (12/20)	70 (14/20)

**Table 4:** Cross-reactivity analysis.

Organism	Titer	Cross-reaction
Human coronavirus 229E	1.4 × 10^5^ TCID50/mL	No
Human coronavirus OC43	1.4 × 10^5^ TCID50/mL	No
Human coronavirus NL63	1.4 × 10^5^ TCID50/mL	No
MERS-coronavirus (irradiated)	1.4 × 10^5^ TCID50/mL	No
Adenovirus	1.4 × 10^5^ TCID50/mL	No
Human metapneumovirus (hMPV)	1.4 × 10^5^ TCID50/mL	No
Human parainfluenza virus 1	1.4 × 10^5^ TCID50/mL	No
Human parainfluenza virus 2	1.4 × 10^5^ TCID50/mL	No
Human parainfluenza virus 3	1.4 × 10^5^ TCID50/mL	No
Human parainfluenza virus 4 a	1.4 × 10^5^ TCID50/mL	No
Human parainfluenza virus 4 b	1.4 × 10^5^ TCID50/mL	No
Influenza A	1.4 × 10^5^ TCID50/mL	No
Influenza B	1.4 × 10^5^ TCID50/mL	No
Enterovirus	1.4 × 10^5^ TCID50/mL	No
Respiratory syncytial virus A	1.4 × 10^5^ TCID50/mL	No
Rhinovirus	1.4 × 10^5^ TCID50/mL	No
*Haemophilus influenzae*	1.0 × 10^6^ cells/mL	No
*Streptococcus pneumoniae*	1.0 × 10^6^ cfu/mL	No
*Streptococcus pyogenes*	1.0 × 10^6^ org/mL	No
Pooled human nasal wash	N/A	No
*Bordetella pertussis*	1.0 × 10^6^ cells/mL	No
*Mycoplasma pneumoniae*	1.0 × 10^6^ cfu/mL	No
*Chlamydia pneumoniae*	1.0 × 10^6^ IFU/mL	No
*Legionella pnuemophila*	1.0 × 10^6^ cfu/mL	No
*Staphylococcus aureus*	>10^4^ cfu/vial	No
*Staphylococcus epidermidis*	1.0 × 10^6^ cfu/mL	No
*Candida albicans*	1.0 × 10^6^ cfu/mL	No

**Table 5:** Potential interfering testing results.

Potential interfering substance	Concentration	Positive results (detected X/3)	Negative results (detected X/3)
Nasal secretion	2.5%	3/3	0/3
	5%	3/3	0/3
	10%	3/3	0/3

Dexamethasone	0.05 mg/L	3/3	0/3
	0.1 mg/L	3/3	0/3
	0.15 mg/L	3/3	0/3

Zanamivir	2.5 mg/L	3/3	0/3
	5 mg/L	3/3	0/3
	10 mg/L	3/3	0/3

Tobramycin	50 mg/L	3/3	0/3
	100 mg/L	3/3	0/3
	150 mg/L	3/3	0/3

Adrenalin	0.1 mg/L	3/3	0/3
	0.2 mg/L	3/3	0/3
	0.25 mg/L	3/3	0/3

Menthol	20 mg/L	3/3	0/3
	25 mg/L	3/3	0/3
	50 mg/L	3/3	0/3

Blood (human)	0.5%	3/3	0/3
	1%	3/3	0/3
	1.5%	3/3	0/3

**Table 6:** Test results compared to DiaCarta QuantiVirus™ SARS-CoV-2.

Flash20 SARS-CoV-2 nucleic acid rapid detection system	DiaCarta QuantiVirus™ SARS-CoV-2		
	Positive	Negative	Total
Positive	131	0	131
Negative	1	83	84
Total	132	83	215
Sensitivity	99.24% (95% CI: 95.85% to 99.98%)		
Specificity	100% (95% CI: 95.65% to 100.00%)		

**Table 7:** Comparison with other COVID-19 nucleic acid system^1)^.

Names	Flash20 SARS-CoV-2 detection system	Xpert Xpress SARS-CoV-2 test	Visby medical COVID-19 point of care test	DiaCarta QuantiVirus™ SARS-CoV-2 Test Kit
Test type	POCT	POCT	POCT	Conventional PCR
Method	RT-PCR	RT-PCR	RT-PCR	RT-PCR
Sample type	Nasopharyngeal and oropharyngeal	Nasopharyngeal, oropharyngeal, nasal, mid-turbinate swab, and nasal wash/aspirate specimens	Nasopharyngeal, anterior nasal, and mid-turbinate swabs	Upper respiratory specimen sputum
Time from sample to answer	30 min	45 min	30 min	>2 hours
LoD	250 copies/mL	0.0200 PFU/mL	435 copies/swab	200 copies/mL or 100 copies/mL^1)^
Sensitivity	98%	97.80%	100%	100%
Specificity	100%	95.60%	95%	100%
Target gene	ORF1ab and N	N2 and E	N	ORF1ab, N, and E

^1)^The LoD of DiaCarta QuantiVirus™ SARS-CoV-2 Test Kit is different in detecting instrument, 200 copies/mL for ABI QuantStudio 5 and ABI 7500 Fast Dx, and 100 copies/mL for Bio-Rad CFX384.

## Data Availability

The data generated or used during the study are available from the corresponding author by request.

## Supplementary Materials

Supplementary Materials Appendix-1: Ct values of LoD estimation study testing. Appendix-2: test results of COVID-19 samples.
