# Supplementary material for: Fast and Sensitive Detection of SARS-CoV-2 Nucleic Acid Using a Rapid Detection System Free of RNA Extraction
Source: Int J Anal Chem. 2023 Jan 20;2023:8053524. doi: 10.1155/2023/8053524 (PMC9883100; doi:10.1155/2023/8053524)
Supplement: Supplementary Materials — Appendix-1: Ct values of LoD estimation study testing. Appendix-2: test results of COVID-19 samples. [file 8053524.f1.docx]

Appendix-1 Ct Values of LoD Estimation Study Testing

| Virus titer copies/mL | ORF1ab Ct (FAM) | RNase P Ct (HEX) | N Ct (ROX) |
| --- | --- | --- | --- |
| 2000 | 21.03 | 17.22 | 20.34 |
| 2000 | 21.04 | 17.1 | 19.82 |
| 2000 | 20.34 | 17.11 | 19.6 |
| 2000 | 20.33 | 17.03 | 19.52 |
| 1000 | 21.25 | 16.67 | 21.3 |
| 1000 | 21.58 | 16.87 | 20.8 |
| 1000 | 21.71 | 17.28 | 20.45 |
| 1000 | 20.92 | 17.47 | 21.07 |
| 500 | 24.35 | 16.84 | 21.95 |
| 500 | 22.23 | 17.34 | 21.09 |
| 500 | 24.45 | 16.84 | 22.05 |
| 500 | 22.56 | 17.07 | 21.37 |
| 400 | 22.54 | 17.25 | 21.88 |
| 400 | 21.81 | 17 | 22.08 |
| 400 | 20.51 | 16.67 | 22.09 |
| 400 | 20.19 | 16.24 | 20.57 |
| 300 | 21.82 | 17.25 | 22.64 |
| 300 | 21.56 | 17.24 | 21.34 |
| 300 | 21.57 | 17.05 | 21.75 |
| 300 | 22.55 | 17 | 22 |
| 200 | 23.71 | 18.02 | N/A |
| 200 | 24.8 | 18.11 | 22.31 |
| 200 | N/A | 18 | N/A |
| 200 | 23.25 | 18.43 | 22.78 |
| 300 | 21.89 | 15.7 | 22.01 |
| 300 | 21.73 | 15.44 | 22.77 |
| 300 | 21.23 | 15.08 | 21.58 |
| 300 | 23.28 | 15.5 | 21.29 |
| 300 | 23.44 | 15.9 | 22.27 |
| 300 | 21.6 | 15.31 | 21.79 |
| 300 | 20.96 | 15.16 | 21.98 |
| 300 | 20.47 | 15.33 | 22.05 |
| 300 | 22.56 | 15.2 | 21.46 |
| 300 | 23.18 | 15.37 | 21.74 |
| 300 | N/A | 15.08 | 23.1 |
| 300 | 23.53 | 15.6 | 21.68 |
| 300 | N/A | 15.56 | 22.33 |
| 300 | 21.33 | 15.18 | 21.36 |
| 300 | 22.76 | 15.33 | 21.17 |
| 300 | 20.11 | 15.52 | 23.68 |
| 300 | 23.33 | 14.93 | 23.13 |
| 300 | 23.94 | 15.18 | 22.18 |
| 300 | 24.14 | 15.3 | 21.2 |
| 300 | 23.39 | 15.66 | 21.43 |
| 250 | 23 | 17.29 | 21.83 |
| 250 | 24.55 | 17.53 | 22.3 |
| 250 | 24.36 | 17.31 | 22.33 |
| 250 | 23.23 | 17.21 | 21.57 |
| 250 | 24.51 | 17.32 | 23 |
| 250 | 23.39 | 17.79 | 22.24 |
| 250 | N/A | 17.52 | N/A |
| 250 | 24.92 | 17.52 | 23.37 |
| 250 | 22.83 | 17.59 | 23.08 |
| 250 | 22.31 | 17.32 | 23.27 |
| 250 | 23.57 | 17.31 | 23.06 |
| 250 | 23.14 | 17.66 | 22.11 |
| 250 | 22.76 | 17.11 | 22.33 |
| 250 | 23.44 | 17.29 | 23.71 |
| 250 | 21.26 | 17.01 | 20.92 |
| 250 | 23.66 | 18.11 | N/A |
| 250 | 26.75 | 17.72 | 28.07 |
| 250 | 24.07 | 17.8 | 22.9 |
| 250 | 23.09 | 17.21 | 23.93 |
| 250 | 20.71 | 17.76 | 21.75 |
| 200 | 24.15 | 17.01 | 22.5 |
| 200 | 23.82 | 17.22 | 22.12 |
| 200 | 23.98 | 16.74 | 22.5 |
| 200 | 23.92 | 16.8 | 23.3 |
| 200 | N/A | 16.88 | 21.54 |
| 200 | 23.89 | 17.02 | 21.81 |
| 200 | 20 | 16.48 | 21.55 |
| 200 | 21.59 | 17.11 | 22.32 |
| 200 | N/A | 17.18 | 22.65 |
| 200 | 22.96 | 17.53 | 22.27 |
| 200 | 26.18 | 18.51 | N/A |
| 200 | 23.31 | 18.28 | 22.82 |
| 200 | 23.71 | 18.02 | N/A |
| 200 | 24.8 | 18.11 | 22.31 |
| 200 | N/A | 18 | N/A |
| 200 | 23.25 | 18.43 | 22.78 |
| 200 | N/A | 18.17 | 23.72 |
| 200 | 21.99 | 17.83 | 23.63 |
| 200 | 26.19 | 18.38 | 22.93 |
| 200 | 20.97 | 18.8 | 22.63 |

Appendix-2 Test Results of COVID-19 Samples

| Patient ID | Flash20 SARS-CoV-2 nucleic acid rapid detection system | | | DiaCarta QuantiVirus™ SARS-CoV-2 Test Kit | |
| --- | --- | --- | --- | --- | --- |
|  | ORF1ab Ct | IC Ct | N Gene Ct | ORF1ab Ct | Rnase P Ct |
| 12-1190 | 22.69 | 15.45 | 22.49 | 25.14 | 21.05 |
| 12-1059 | N/A | 14.63 | N/A | No Ct | 26.98 |
| 12-1060 | N/A | 15.64 | N/A | No Ct | 29.93 |
| 12-1067 | N/A | 17.08 | N/A | No Ct | 28.5 |
| 12-1068 | N/A | 15.8 | N/A | No Ct | 26.87 |
| 12-1069 | N/A | 17.55 | N/A | No Ct | 29.46 |
| 12-1070 | N/A | 16.86 | N/A | No Ct | 26.14 |
| 12-1071 | N/A | 16.41 | N/A | No Ct | 27.07 |
| 12-1072 | N/A | 18.35 | N/A | No Ct | 26.55 |
| 12-1073 | N/A | 16.21 | N/A | No Ct | 30.62 |
| 12-1074 | N/A | 17.32 | N/A | No Ct | 29.56 |
| 12-1076 | N/A | 19.57 | N/A | No Ct | 27.99 |
| 12-1077 | N/A | 16.26 | N/A | No Ct | 26.58 |
| 12-1078 | N/A | 17.46 | N/A | No Ct | 28.92 |
| 12-1079 | N/A | 18.02 | N/A | No Ct | 26.01 |
| 12-1080 | N/A | 17.37 | N/A | No Ct | 27.51 |
| 12-1081 | N/A | 17.61 | N/A | No Ct | 21.51 |
| 12-1082 | N/A | 12.56 | N/A | No Ct | 27.81 |
| 12-1083 | N/A | 20.21 | N/A | No Ct | 29.55 |
| 12-1084 | N/A | 14.16 | N/A | No Ct | 28.73 |
| 12-1085 | N/A | 13.36 | N/A | No Ct | 28.92 |
| 12-1086 | N/A | 16.16 | N/A | No Ct | 27.86 |
| 12-1087 | N/A | 13.87 | N/A | No Ct | 30.26 |
| 12-1088 | N/A | 16.12 | N/A | No Ct | 28.86 |
| 12-1089 | N/A | 13.84 | N/A | No Ct | 25.91 |
| 12-1090 | N/A | 14.91 | N/A | No Ct | 24.89 |
| 12-1091 | N/A | 15.59 | N/A | No Ct | 27.74 |
| 12-1092 | N/A | 17.14 | N/A | No Ct | 27.43 |
| 12-1093 | N/A | 16.76 | N/A | No Ct | 29.66 |
| 12-1094 | N/A | 12.82 | N/A | No Ct | 27.39 |
| 12-1095 | N/A | 10.05 | N/A | No Ct | 27.33 |
| 12-1360 | 17.3 | 11.15 | 17.98 | 20.98 | 20.65 |
| 12-1391 | 19.02 | 11.91 | 19.35 | 24.17 | 22.25 |
| 12-1431 | 20.04 | 14.1 | 23.09 | 33.77 | 18.94 |
| 12-1454 | 20.02 | 12.5 | 21.58 | 24.03 | 19.9 |
| 12-1455 | 20.09 | 15.05 | 21.52 | 22.07 | 19.61 |
| 12-1501 | 11.5 | 13.71 | 11.83 | 16.55 | 18.09 |
| 12-1506 | 15.2 | 14.3 | 16.36 | 20.1 | 3.88 |
| 12-1509 | 19.88 | 18.22 | 20.83 | 32.9 | 27.64 |
| 12-1511 | 18.2 | 16.2 | 22.07 | 27.61 | 27.4 |
| 12-1729 | 18.67 | 14.76 | 19.6 | 25.49 | 24.04 |
| 12-1809 | 20.97 | 11.46 | 20.53 | 30.21 | 21.17 |
| 12-1811 | 13.94 | 12.64 | 14.63 | 17.77 | 20.1 |
| 12-1832 | 17.81 | 12.3 | 17.36 | 22.24 | 17.25 |
| 12-2067 | 14.61 | 12.52 | 11.2 | 17.32 | 20.51 |
| 12-2068 | 24 | 10.71 | N/A | 35.82 | 22.91 |
| 12-2069 | 17.86 | 13.2 | 21.03 | 28.51 | 21.42 |
| 12-2091 | 19.28 | 12.83 | 19.65 | 23.32 | 21.98 |
| 12-2122 | 16 | 12.52 | 15.51 | 25.9 | 21.86 |
| 12-2142 | 24.07 | 14.04 | 22.03 | 29.65 | 22.9 |
| 12-2145 | 20.06 | 14.46 | 19.14 | 24.81 | 21.62 |
| 12-2221 | 11.57 | 12.19 | 11.18 | 16.04 | 18.43 |
| 12-2222 | 16.24 | 11.97 | 15.37 | 18.49 | 20.5 |
| 12-2223 | 11.64 | 14.45 | 12.14 | 17.01 | 5.23 |
| 12-2397 | 17.96 | 14.1 | 16.27 | 21.66 | 22.31 |
| 12-2444 | N/A | 11.07 | N/A | 34.03 | 22.07 |
| 12-2446 | 11.18 | 11.82 | 10.51 | 16.7 | 19.68 |
| 12-2447 | 12.75 | 15.24 | 11.32 | 16.58 | 19.96 |
| 12-3152 | 17.69 | 13.25 | 17.58 | 25.66 | 23.73 |
| 12-3229 | 18.62 | 9.4 | 19.55 | 27.35 | 22.9 |
| 12-3232 | 18.36 | 11.9 | 17.03 | 23.37 | 22.74 |
| 12-3235 | 15.22 | 17.86 | 12.91 | 16.31 | 18.87 |
| 12-3236 | 14.64 | 16.18 | 13.3 | 18.16 | 22.22 |
| 2101250298 | 22.15 | 12.9 | 18.76 | 32.77 | 29.06 |
| 2101110009 | 16.13 | 15.13 | 16.46 | 27.89 | 25.36 |
| 2101110289 | 21.46 | 14.33 | 20.55 | 27.44 | 30.21 |
| 2101110293 | 15.6 | 16.2 | 15.86 | 32.36 | 30.93 |
| 2101110506 | 16.64 | 15.44 | 15.33 | 28.53 | 32.4 |
| 2101150027 | 21.64 | 12.75 | 21.4 | 32.84 | 31.12 |
| 2101150076 | 25.24 | 11.86 | 22.36 | 33.86 | 27.39 |
| 2101200099 | N/A | 14.19 | N/A | No Ct | 27.82 |
| 2101200145 | N/A | 13.54 | N/A | No Ct | 26.15 |
| 2101200233 | N/A | 14.5 | N/A | No Ct | 27.23 |
| 2101200259 | N/A | 11.65 | N/A | No Ct | 23.82 |
| 2101200298 | N/A | 13.45 | N/A | No Ct | 28.05 |
| 2101200322 | N/A | 14.44 | N/A | No Ct | 29.49 |
| 2101200350 | N/A | 15.61 | N/A | No Ct | 30.43 |
| 2101200361 | N/A | 17.34 | N/A | No Ct | 28.99 |
| 2101200472 | N/A | 13.2 | N/A | No Ct | 28.1 |
| 2101210026 | N/A | 13.37 | N/A | No Ct | 24.1 |
| 2101210047 | N/A | 11.58 | N/A | No Ct | 25.89 |
| 2101210048 | N/A | 15.05 | N/A | No Ct | 28.5 |
| 2101210060 | N/A | 14.95 | N/A | No Ct | 26.22 |
| 2101210082 | N/A | 13.27 | N/A | No Ct | 26.45 |
| 2101230023 | N/A | 14.95 | N/A | No Ct | 27.59 |
| 2101230034 | N/A | 17.25 | N/A | No Ct | 30.58 |
| 2101230046 | N/A | 12.22 | N/A | No Ct | 25.53 |
| 2101230085 | N/A | 12.91 | N/A | No Ct | 25.54 |
| 2101250020 | 17.51 | 13.93 | 18.84 | 34.35 | 32.69 |
| 2101250027 | 22.1 | 18.86 | N/A | 31.85 | 30.96 |
| 2101250161 | N/A | 17.52 | 22.83 | 34.81 | 31.02 |
| 2101250242 | 17.68 | 11.63 | 18.57 | 29.17 | 30.01 |
| 2101250272 | 19.45 | 15.5 | 19.11 | 32.9 | 30.18 |
| 2101270753 | N/A | 14.24 | N/A | No Ct | 26.24 |
| 2101270776 | N/A | 14.49 | N/A | No Ct | 27.71 |
| 2101270787 | N/A | 16.52 | N/A | No Ct | 29.76 |
| 2101270788 | N/A | 17.26 | N/A | No Ct | 27.49 |
| 2101270798 | N/A | 14.94 | N/A | No Ct | 28 |
| 2101270810 | N/A | 15 | N/A | No Ct | 28.69 |
| 2101270811 | N/A | 13.94 | N/A | No Ct | 28.28 |
| 2101270816 | N/A | 13.27 | N/A | No Ct | 27.03 |
| 2101270821 | N/A | 12.16 | N/A | No Ct | 25.55 |
| 2101270824 | N/A | 12.26 | N/A | No Ct | 26.68 |
| 2101270825 | N/A | 14.28 | N/A | No Ct | 28.2 |
| 2101270827 | N/A | 12.72 | N/A | No Ct | 27.15 |
| 2102060153 | 20.5 | 16.42 | 20.16 | 36.32 | 29.59 |
| 2102060161 | 16.83 | 11.55 | 18.2 | 29.51 | 29.3 |
| 2102060187 | 11.24 | 10.5 | 12.84 | 28.61 | 26.02 |
| 2102060274 | 11.73 | 13.03 | 9.86 | 28.19 | 23.25 |
| 2102060278 | 14.43 | 16.13 | 13.9 | 28.36 | 25.02 |
| 2102100700 | 18.32 | 14.13 | 18.09 | 30.86 | 29.89 |
| 2102120463 | 20.42 | 10.77 | 18.88 | 33.97 | 27.15 |
| 2102120486 | 22.26 | 10.9 | N/A | 34.55 | 26.27 |
| 2102120497 | 19.32 | 14.84 | N/A | 35.34 | 28.16 |
| 2102120621 | 11.8 | 13.01 | 15.42 | 30.96 | 26.01 |
